# Bone defect reconstruction using Masquelet technique for calcaneal chondroblastoma: a case report

**DOI:** 10.1093/jscr/rjad401

**Published:** 2023-07-14

**Authors:** Xianwei Chen, Gong Chen, Zhifu Chen, Jing Zhang

**Affiliations:** Department of Orthopedics, Cancer Hospital of Yunnan Province, The Third Affiliated Hospital of Kunming Medical University, Kunming, China; Department of Orthopedics, Cancer Hospital of Yunnan Province, The Third Affiliated Hospital of Kunming Medical University, Kunming, China; Department of Orthopedics, Cancer Hospital of Yunnan Province, The Third Affiliated Hospital of Kunming Medical University, Kunming, China; Department of Orthopedics, Cancer Hospital of Yunnan Province, The Third Affiliated Hospital of Kunming Medical University, Kunming, China

## Abstract

Masquelet technique demonstrated superiority in reconstructing long bone defect after trauma or infection. However, reports in foot tumor were rare. A 24-year-old male diagnosed with calcaneal chondroblastoma who had a defect of calcaneal after intralesional curettage. We reconstructed the defect by Masquelet technique. This is the first case as far as we know that reported Masquelet technique for calcaneal tumor. The technique to treat irregular bone defects after operation can be considered in other similar situations.

## INTRODUCTION

Chondroblastoma is a benign cartilaginous neoplasm of bone that will most frequently appear in the calcaneus when found in the foot. Calcaneal chondroblastoma makes up around 10% of all cases with higher recurrence than other locations [1].The bone lesion is typically osteolytic, which is often accompanied with cortical erosion. After curettage surgery, bone defect is often serious. The osseous integration with the graft is difficult to achieve because of poor local blood supply of calcaneus.

Masquelet technique is a recognized method for repairing large-area bone defect, which was first proposed by Masquelet [2]. The technique demonstrated superiority in reconstructing long bone defect after trauma or infection, but its safety and validity in foot tumor were not clear. Here we report a case of using Masquelet technique to reconstruct bone defect following curettage of calcaneal chondroblastoma.

## CASE REPORT

**Figure 1:**
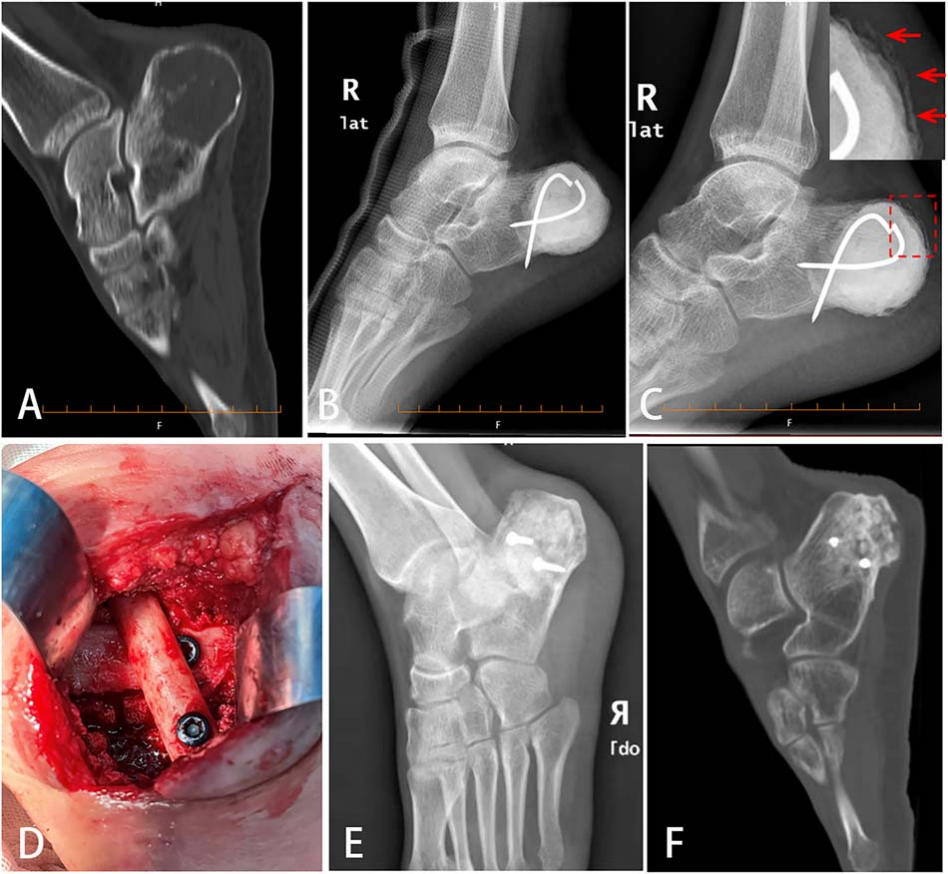
Bone defect reconstruction using Masquelet technique for calcaneal chondroblastoma. (**A**) X-ray showed osteolytic bone lesion located in posterior of right calcaneus body. The cortex was thinned because of tumor aggression. (**B**) After tumor was removed totally, bone cement (PMMA) was filled into the cavity and fixed with two cross Kirschner wires. (**C**) Six weeks later, the induced membrane on the calcaneal surface could be seen obviously (indicated by arrow). (**D**) Autologous fibular segment shoring and bone graft were filled into the cavity to reconstruct the defect. (**E**，**F**) Eleven months later, DR (E) and CT (F) scan showed good bone healing.

A 24-year-old man complained of pain in the right heel for more than 2 years without any trauma history. The right heel had mild swelling, and movement of ankle was slightly limited because of mild contracture of the Achilles tendon. X-ray of right foot showed low-density lesion on the posterior of calcaneus. CT scan showed an osteolytic destruction area of 4.1 × 3.0 × 3.9 cm with clear border and slight cortical discontinuity (Fig. 1A). The Musculoskeletal Tumor Score and American Orthopedic Foot and Ankle Society Hind Foot Score of this patient were 22 and 82 points, respectively, before surgery. Biopsy was performed and the pathological results showed benign tumor of chondroblastoma possibly. Ethical approval for this study was obtained from hospital Ethics Committee and the informed consent for the surgery was signed with the patient.

We choose Masquelet technique surgery for this patient. First, calcaneal tumor was totally removed by intralesional curettage. Local adjuvants included high-speed burring and 95% alcohol inactivation for 5 mins. Cortex defect of calcaneal was about 8 cm in longest diameter. Bone cement (PMMA) spacer was packed into the defect and fixed with two crossed Kirschner wires (Fig. 1B). Six weeks later, X-ray showed induced membrane around the bone cement surface (Fig. 1C). Second, bone cement was removed. Crossed autologous fibular segments (splitted in the middle) shoring with two screws and allogeneic cancellous graft associated with calcium phosphate and bone morphogenetic proteins (BMPs) were filled into the cavity to reconstruct the bone defect (Fig. 1D). The induced membrane wrapped the bone graft completely.

The patient’s postoperative recovery was good. The wound healed up in 2 weeks with no rejection reaction. Six weeks after surgery, the patient was able to walk with the help of single crutch. Three months later, the patient could walk by himself and ankle movement returned to normal. Six months later, the patient could walk freely without pain. Eleven months later, X-ray and CT scan showed no tumor relapse and good bone healing of calcaneus (Fig. 1E and F). The Musculoskeletal Tumor Score and American Orthopedic Foot and Ankle Society Hind Foot Score were up to 30 and 90 points, respectively. We have obtained the approval of the Ethics Committee and the written informed consent from patient before operation.

## DISCUSSION

There are various methods of bone reconstruction of postoperative bone defects, such as vascularized fibular graft, Ilizarov technique, bulk allograft and so on. Vascularized fibular graft has risks of infection, fracture and postoperative vascular occlusion [3]. Because of the long treatment time and inconvenient life for patients, Ilizarov technique is generally used to repair bone defects of long bone within 6 cm, and it easily develops infection [4]. Bulk allograft is often difficult to avoid bone nonunion and immune rejection, especially in the calcaneus site with poor blood supply. The Masquelet technique has a significant superiority to traditional methods. It is simple to operate without restrictions on tumor location or defect shape. Bone cement spacer could kill residual tumor cells by powerful exothermic reaction. The induced membrane could cover the bone graft perfectly and promote bone healing [5].

The induced membrane is composed of epithelioid cells, fibroblasts, myofibroblasts and type I collagen, which is rich in blood vessels and nourishes the bone defect area. Mesenchymal stem cells were also found in the induced membrane, indicating that the membrane has potential to differentiate into osteogenic and chondrogenic cells [6]. It also secretes BMP-2, VEGF and TGF to promote blood vessel formation and bone remodeling [7]. For this patient, although the cortex defect was more than one-third of whole calcaneus following curettage, the calcaneal-talar joint was still intact. The residual calcaneus was partially stable. So we choose autologous fibular segments shoring and cancellous bone graft to reconstruct bone defect without internal fixation.

The right timing of weight bearing after graft implantation depend on many factors. For trauma with defects of the foot and ankle, it was recommended to avoid weight bearing altogether at this stage. Touch-toe weight bearing with a frame or crutches was mobilized for 6 weeks [5]. For calcaneal tumor, the timing of weight bearing after graft implantation was delayed because of adjuvant chemotherapy, which might inhibit the osseous integration with the graft [8]. For this patient of benign tumor, chemotherapy was not required and the residual calcaneus was stable reconstructed. We advised him touch-toe weight bearing with the help of single crutch in 6 weeks after graft implantation. Three months later, formation of bony callus was confirmed by X-ray examination. The patient could walks independently and weight bearing altogether was good for bone remodeling.

## CONCLUSION

In conclusion, we successfully reconstructed bone defect using Masquelet technique following curettage in a calcaneal chondroblastoma patient. We provide a new reconstruction choice for bone defect after surgery of calcaneal tumors. The safety and validity of Masquelet technique is worth trying in future by more clinical cases.

## Data Availability

All data and materials can be provided if necessary.
